# *Givira ethela* (Neumoegen and Dyar, 1893) (Lepidoptera: Cossidae), A Previously Unidentified Pest on *Vitis vinifera* (L.)

**DOI:** 10.3390/insects12030239

**Published:** 2021-03-12

**Authors:** Davide Scaccini, Enrico Ruzzier, Kent M. Daane

**Affiliations:** 1 Department of Agronomy, Food, Natural Resources, Animals and the Environment (DAFNAE), University of Padova, Viale dell’Università 16, Legnaro, I-35020 Padova, Italy; 2 Department of Environmental Science, Policy, & Management, University of California Berkeley, Mulford Hall, Berkeley, CA 94720, USA; kdaane@ucanr.edu

**Keywords:** carpenter millers, carpentermoth, grape, moth, viticulture, vineyard, wood borer, new pest, mealybug

## Abstract

**Simple Summary:**

In California, grape cultivation for wine, juice, fresh and raisin markets is one of the most profitable agricultural sectors. Pathogens, weeds and insect pests cause millions in crop loss and result in additional costs through the use of pesticides and other control tools. Here, we report on the emergence of a new California grape pest, the carpentermoth *Givira ethela* (Neumoegen and Dyar, 1893). This paper also aimed at the moth’s identification through molecular and morphological features and updates the distribution of *G. ethela* in California. Furthermore, the presence of *G. ethela* larval galleries appears to facilitate the mealybug pest *Planococcus ficus* Signoret, 1875, providing better access to vine sap and protection from natural enemies, environmental stresses, and pesticide treatments. We propose that management practices against *G. ethela* should include the correct identification of the pest and of its damage, but also the investigation of monitoring methods, economic thresholds, biological controls, and a better understanding of the relationship between *G. ethela* and *P. ficus* or other mealybug pest species.

**Abstract:**

Grape cultivation is a billion-dollar agricultural sector in California, where invasive or novel pest species can disrupt management practices. We report herein on a new pest associated with California vineyards, the carpentermoth *Givira ethela* (Neumoegen and Dyar, 1893). Rather than an invasive species, *G. ethela* appears to be a newly recognized wood-boring pest of *Vitis vinifera* (L.) in regions of California’s Central Valley, where its initial occurrence has been dated back to, at least, the beginning of the 2000s. The habitus of adult, genitalia and pupa is illustrated. *Givira ethela* distribution in California is updated including published records and new data. Carpentermoth galleries seem to facilitate the access of *Planococcus ficus* Signoret, 1875 to vine sap and protection from natural enemies, environmental stresses, and pesticide treatments. Notes on pest status, life history, monitoring practices, natural enemies, and management options on grapes are also discussed. Tools for the Integrated Pest Management of *G. ethela* should include the correct identification of the insect and its damage, a full understanding of its biology and ecology, the application of monitoring methods, and the identification of economic thresholds and injury levels.

## 1. Introduction

Wood boring beetles and moths can damage cultivated crops, particularly fruit and ornamental trees [1]. Among moths, species in the Cossidae, Hepialidae, Sesiidae and Xyloryctidae are widely recognized as having larvae that are wood boring pests on tree crops, where they feed upon both the vascular and/or structural tissues of the plant, e.g., [2,3,4]. The trophic activity of these larvae can cause a reduction in structural stability of the host plant, and direct plant-stress, and may promote the establishment of phytopathogens that further damage plant growth or crop yield, e.g., [1,5,6,7,8,9].

Among the abovementioned families, there are at least five cossid and two sesiid species that are reported to cause damage in vineyards worldwide [5,7,10,11]. Reports of carpentermoth larvae damage to grapevines most commonly refer to the cossid *Cossus cossus* (Linnaeus, 1758) [12], which has a long history as a polyphagous pest in southern Europe vineyards and orchards and often requires the application of cultural or chemical management practices [1,13]. Other known wood boring cossids include *Paropta paradoxus* (Herrich-Schäffer, 1851) that was recognized as grapevine pest in Israel and, more recently, in Turkey [14,15] and *Polyphagozerra coffeae* (Nietner, 1861), a polyphagous pest, feeding on more than 50 plant species, and reported to cause severe damage to grapevines in Taiwan when high population densities were left unchecked [16]. In the 1980s, the cossid *Coryphodema tristis* (Drury, 1782) was documented to feed on and damage mature (>1-yr-old) grapevine wood in South Africa and was also associated with rot fungi that further damaged the vine [8]. More recently, the carpentermoth *Dervishiya cadambae* (Moore, 1865) (Lepidoptera: Cossidae) was found boring in both the sapwood and heartwood of grapevine trunks in India, reducing vine vitality and productivity [5] and joining the list of grapevine wood boring pests.

The aim of this paper is to report on a new wood boring carpentermoth infestation on grapevines in California’s San Joaquin Valley. Worldwide, grape value exceeds $68 billion (USD) with about 7.1 million ha in production [17]. In California, wine, juice, fresh and dried grapes are a $5 billion enterprise with about 371,500 ha in production and a leading sector in Californian agriculture [18]. Therefore, novel insect pests, weeds and pathogens that require additional management cost and/or pesticide applications are a concern for vineyard managers and hamper the development of sustainable management practices [19,20]. Here, the habitus of adult, genitalia and pupa of a carpentermoth found causing considerable damage to grapevines is illustrated. The carpentermoth populations appear to be confined to regions in the San Joaquin Valley, in the middle of the state, after 20 years since its first detection. Its distribution in California is here updated including published records and new data. We also observed an association between carpentermoth galleries and mealybugs, and discuss the mutualistic association between the cossid larvae and mealybugs. Finally, notes on pest status, life history, monitoring practices, natural enemies, and management options applicable for carpentermoth control on grapevine are discussed.

## 2. Materials and Methods

### 2.1. Insect Collections and Rearing

Carpentermoth infestations on grapevines in California were first noted in 2002 on older (>20-yr-old) raisin vineyards in Fresno County, California (USA), and later tentatively identified as a *Givira* species by Dr. Jerry Powell (University of California, Berkeley, CA, USA). To determine the extent of infestations, surveys were conducted sporadically from 2005 to 2007 in vineyards known to be infested at four sites: (1) Reedley (36.5749, −119.4261, 100 m a.s.l.), (2) Kingsburg (36.5110, −119.5380, 90 m a.s.l.), (3) Parlier (36.5998, −119.5075, 100 m a.s.l.), and (4) Clovis (36.7999, −119.6177, 115 m a.s.l.). Three of these vineyards were *cv*. Thompson seedless managed for raisin or wine production, and one vineyard was a *cv*. Scarlet Royal managed for fresh table grapes. All blocks were mature (>15-yr-old) and had a history of mealybug, *Planococcus ficus* Signoret, 1875 (Hemiptera: Pseudococcidae) infestations that were treated with 1–2 annual applications of pesticides (imidacloprid, buprofezin, or spirotetramat). In these initial samples, vine bark was peeled back, and sections of the trunk and cordon were searched for carpentermoth galleries. The number of vines with active carpentermoth galleries and the number of galleries with *P. ficus* were recorded.

In 2018, the Parlier and Clovis sites were more systematically sampled, in order to collect specimens for species identification. Samples were taken on 1 and 14 August at the Parlier site, and 20 August and 30, 31 July at the Clovis site. Collections were made by removing the bark on vine trunks and cordons, with each vine searched for 30 min. During each sample date, 10–25 vines were sampled, split between two different rows in each vineyard. Once active carpentermoth larvae were found, the trunk material surrounding the moth larvae were excised using wood chisels to remove the larvae as well as a portion (4 × 4 cm) of wood surrounding the larvae to collect live and undamaged larvae. In the laboratory, collected larvae were placed singly in a vial provisioned vine wood slivers; after which each vial was checked periodically and received honey-water as additional food and moisture. A few drops of honey-water were added twice a week directly on wood slivers in the vial using a plastic pipette. Larvae and pupae were then reared to adults under laboratory conditions (26 ± 2 °C, 65 ± 5% RH, 16:8 L:D), and freshly eclosed adults were killed in jars with ethyl acetate vapors and pinned for species identification.

### 2.2. Genitalia Preparation

Genitalia dissection and microscopic slide preparation of the abdomen was performed following methodologies illustrated by Timossi and Ruzzier [21], adapted from Clarke [22] and Hardwick [23]. After its detachment, the abdomen was macerated in boiling 5% KOH solution for 15–20 min, cleaned with distilled water with a few drops of glacial acetic acid, and then stained with chlorazol black. After this preparation, genital parts were dissected and cleaned in 50% ethanol, dehydrated in absolute ethanol, and finally embedded in euparal. For microscopic slide imaging, a Nikon Eclipse E100 microscope was used, equipped with a Sony Colour CCD 5.1 Mp TP 5100 micro-camera with X-Entry software.

### 2.3. DNA Barcoding

DNA extraction and purification were conducted on five adults (one dry leg each) and two larvae (whole insects), following the salting-out procedure [24]. A partial region of the cytochrome c oxidase subunit I (COI) gene was amplified with primers HCO2198 (5′-GGTCAACAAATCATAAAGATATTGG-3′) and LCO1490 (5′-TAAACTTCAGGGTGACCAAAAAATCA-3′) [25]. PCR products were purified using Exonuclease and Antarctic Phosphatase (GE Healthcare, Wauwatosa, WI, USA) and sequenced at the BMR Genomics Service (Padua, Italy). Sequences were edited and aligned using MEGA X [26], and subsequently translated with Transeq [27] to exclude the presence of stop codons in the coding region. A GenBank BLAST analysis of the sequences obtained was run through the NCBI website [28] and the integrated bioinformatics platform Barcode of Life Data (BOLD) System database [29] was used to assess the identity of the sequences.

### 2.4. Phylogenetic Analysis

To produce the most complete COI dataset, barcodes obtained were integrated with the complete sequences available in BOLD System ([29], see Table S1). The cossid species *Hypopta palmata* Barnes and McDunnough, 1910 and *C. cossus* were included in the analysis as outgroups. Evolutionary analysis was conducted in MEGA X [26] and the phylogeny was inferred by using the Maximum Likelihood (ML) method and Tamura-Nei model [30], with 1000 bootstrap replications. The Neighbor-Joining (NJ) method was also inferred for comparison, with 1000 bootstrap replications. The pairwise genetic distances between sequences were calculated using MEGA X [26], under default settings.

### 2.5. Statistical Analysis

Results are presented as mean ± SE. The 2018 data were analyzed to test carpentermoth infestation levels using generalized linear mixed model with the MIXED procedure of SAS (ver. 9.4) [31]. Treatment “site” (Parlier vs. Clovis) and the interaction “row*site” were considered as independent variables, while the survey event (two surveys per site) was included as random factor. The effect of treatments on infestation of carpentermoth larvae was tested using an F test (α = 0.05) followed by the Tukey–Kramer test as post hoc for comparisons (α = 0.05). Prior to the analyses data were log (n + 1) transformed to meet ANOVA assumptions.

## 3. Results

### 3.1. Molecular Identification

**Figure 1:**
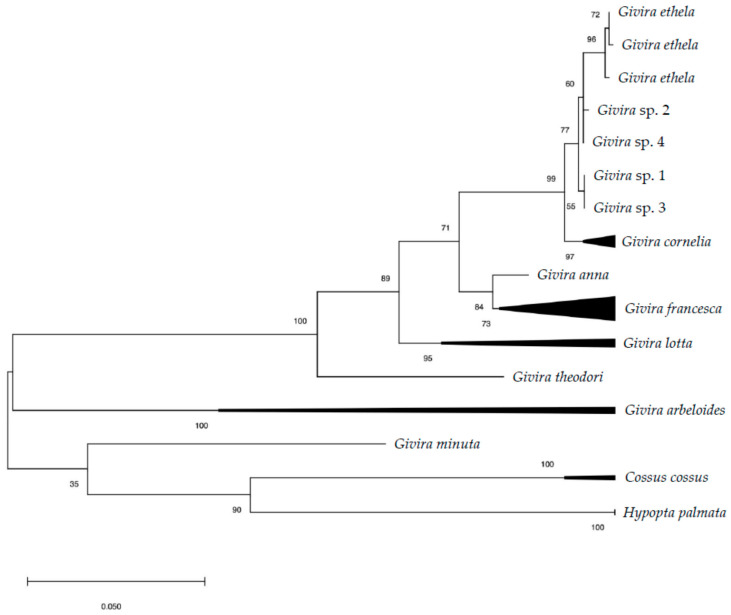
Maximum likelihood (ML) tree based on COI sequences of selected North American *Givira* species. Numbers on the nodes refer to ML bootstrap values. *Hypopta palmata* and *Cossus cossus* are included as outgroups.

DNA barcoding resulted in four barcodes (three adults and one larva) of 621 bp with an overall distance of 0.2 ± 0.1% (Table S2). Both analyses conducted in GeneBank and BOLD System returned a mean similarity >99% with *Givira ethela* (Neumoegen and Dyar, 1893) (Lepidoptera: Cossidae). The phylogenetic reconstruction, inclusive of selected representatives of *Givira* Walker, 1856 (Hypoptinae), *Hypopta* Hübner, 1818 (Hypoptinae) and *Cossus* Fabricius, 1793 (Cossinae), resulted in a consensus tree in which *G. ethela* clusters together with *Givira cornelia* (Neumoegen and Dyar, 1893) with high bootstrap support (ML 99%; NJ 100%) and a genetic distance of 1.3%. The *G. ethela* cluster included seven sequences and had medium-high support (ML 77%; NJ 81%), while the *G. cornelia* grouped six sequences with high support (ML 97%; NJ 99%; Figure 1).

### 3.2. Morphological Identification and Related Features

**Figure 2:**
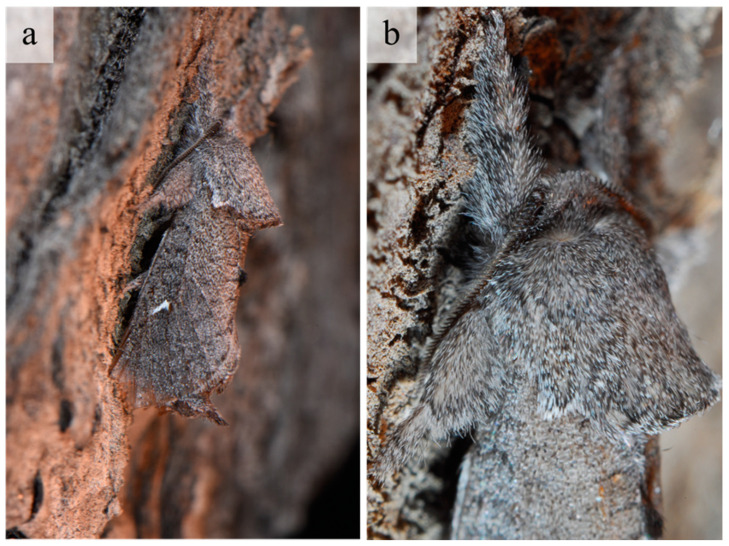
*Givira ethela* adult. (**a**) On the vine bark; (**b**) Head and prothorax magnification.

**Figure 3:**
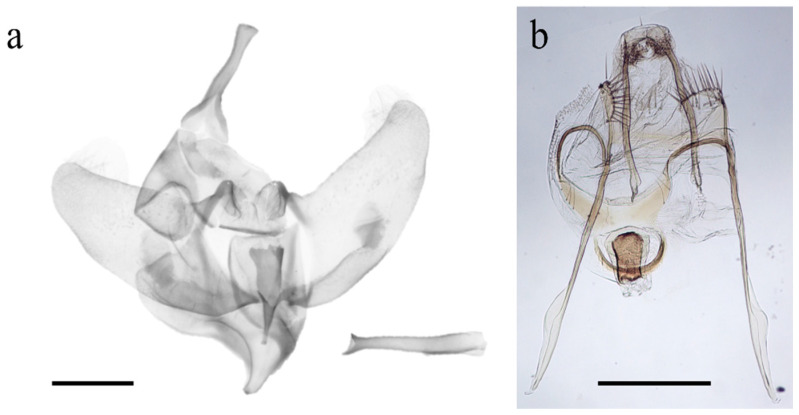
Genitalia of *Givira ethela*. (**a**) Complete male genitalia and aedeagus; (**b**) Female genitalia. Scale bar = 1.0 mm.

**Figure 4:**
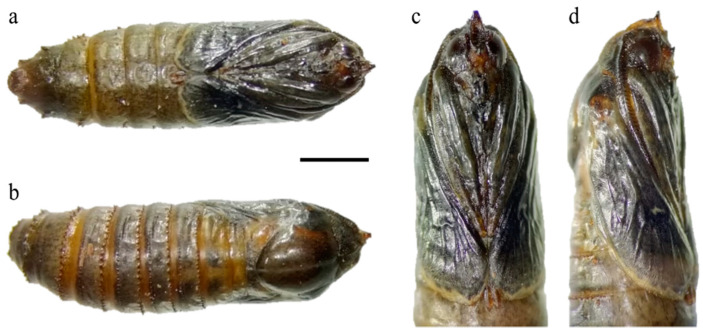
Pupa of *Givira ethela*. (**a**,**c**) Frontal view, at different magnifications; (**b**) Back side; (**d**) Right side. Scale bar: (**a**,**b**) = 2.0 mm; (**c**,**d**) = 1.5 mm.

The external habitus of the reared specimens fall under *G. ethela*, following the identification key provided by Barnes and McDunnough [32] and matching the morphological traits reported in Neumoegen and Dyar [33] and Dyar [34], and illustrated in Seitz [35]. *Givira ethela* is a dark colored moth with grayish–brownish body hairs and scales; adults present a whitish ✓-shaped mark on the forewing (Figure 2). Male and female genitalia are reported in Figure 3 for illustrative purposes only. The pupa shows rows of spine-like processes on abdominal segments (Figure 4).

### 3.3. Distribution and Damage Assessment

**Figure 5:**
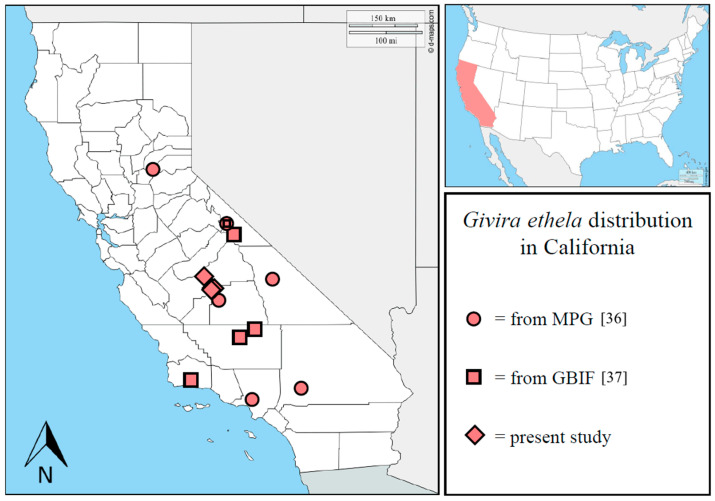
Updated distribution of *Givira ethela* in California. Adapted from www.d-maps.com. Data from MPG [36] and GBIF [37] are included.

Including previous published records and present data, the distribution of *G. ethela* in California is here updated, and it is recorded in nine different counties (Figure 5).

**Figure 6:**
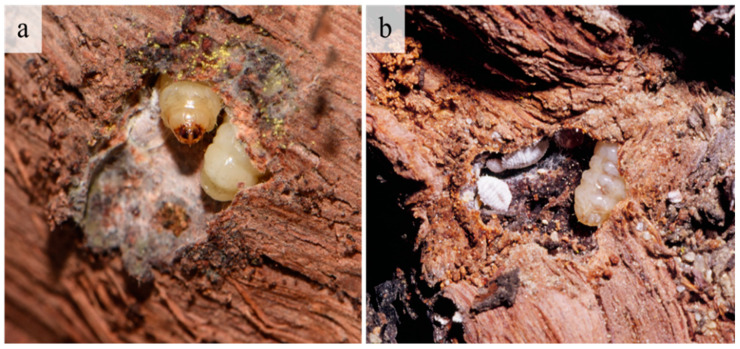
Galleries and *Givira ethela* larvae in grapevine trunks. (**a**) A mature larva, with silk inside the gallery; (**b**) Larva with different stages of the mealybug *Planococcus ficus*.

A monitoring program for *G. ethela* is not currently developed; however, without peeling bark, signs of an infestation are evident through the presence of emerging exuviae on trunks, and frass or silk at the entrance of galleries or on the bark of highly infested vines. Still, the primary damage is under the bark where the larvae create feeding galleries in the cambium layer (Figure 6).

From the 2018 field surveys, 361 larvae were collected, 172 in Parlier and 189 in Clovis. Infestation levels did not differ between the two sites (F_1, 80_ = 0.50, *p* = 0.4814), while significant differences were seldom observed among rows of the same field in the “row*site” interaction (F_4, 80_ = 2.76, *p* = 0.0334). Infestation level ranged between 0 to 37 larvae per vine. Seven living pupae were collected on 20 and 30 July, and on 1 August 2018. Some of the larvae collected in 2018 pupated soon after being moved to the laboratory, where they eclosed in late August–September.

**Figure 7:**
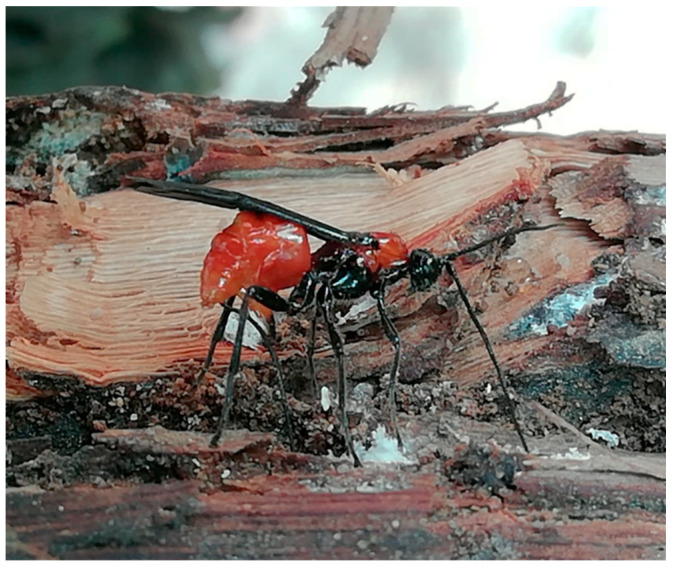
A *Digonogastra* sp. ovipositing through the bark of a *Givira ethela* infested vine (Clovis site, August 2018).

Often associated with the *G. ethela* galleries were different stages of the mealybug *P. ficus*, frequently aggregated in tunnels developing just under the grapevine bark (Figure 6). Several *Digonogastra* sp. (Hymenoptera: Braconidae) were reared from *G. ethela*, and in mid to late summer adult *Digonogastra* were commonly observed searching for and parasitizing carpentermoth in the infested vineyard (Figure 7).

## 4. Discussion

The carpentermoth *G. ethela* is here reported as pest on mature grapevines in California’s San Joaquin Valley. Pest occurrence is estimated to the beginning of 2000s, when attacks by a carpentermoth were first observed, and almost as an oddity the carpentermoth remained unidentified until this work. In our preliminary surveys of vineyards, pest presence appears to be scattered rather than clustered throughout the monitored vineyards. We note that our surveys were conducted in vineyards known to be infested by *G. ethela* for many years and there is no information on how widespread this pest is throughout this region or in other California grape growing areas. Vasquez et al. [38] noted moth larvae feeding on vines in Fresno County in 2010 and are probably referring to this pest. From our limited survey, *G. ethela* attacked table, raisin and juice grapes, each with some level of insecticide applications targeting *P. ficus*. The infested vineyards were mature (>20-yr-old) and we suspect this age preference may be associated with the greater size of the trunks, richer in phloem and better capable to support the development of multiple larvae for several years. Indeed, *Givira* larvae are known to feed on the phloem of their host [39], and other carpentermoth species larvae usually prefer to growth in old grapevine trunks [14].

*Givira ethela* adults are dark colored and have grayish-brownish body hairs and scales. Quoting Metzler [40], most (11 out of 15) of the North American *Givira* species are dark colored, including *G. ethela*, while *G. carla* Dyar, 1923, *G. cornelia*, *G. durangona* (Schaus, 1901), and *G. delindae* Metzler, 2017 are extensively white with few or no dark markings. According to Clench [41], *G. ethela* has some similarities for external characters with *Givira leonera* Clench, 1957, a Neotropical moth described from Chile.

In the phylogenetic reconstruction, *G. ethela* clusters with *G. cornelia* with an overall mean distance of 1.3%. *Givira cornelia* occurs in sympatry with *G. ethela* for most of its range [32,42,43], and further morphological and barcoding data are required to shed light about the relation between the two species. Although *G. ethela* and *G. cornelia* cluster separately in our analyzes, the low genetic diversity and the robustness of their clade (ML 99%; NJ 100%) would seem to indicate their belonging to a single variable taxon. However, in anticipation of a possible taxonomic rearrangement, our specimens remain attributable to the current interpretation of *G. ethela*.

*Givira* includes species distributed in the Nearctic and most in the Neotropics [44]. At least 15 species occur in the United States, mostly distributed in the southwestern part of the country [32,45], including pest species as *G. lotta* Barnes and McDunnough, 1911, a wood boring carpentermoth attacking ponderosa pines [46]. *Givira ethela* is present in Colorado (type locality), California, Nevada, and Utah [32,34,36,37]. The biology of this species is almost unknown, with only Antelope Bitterbrush, *Purshia tridentata* (Pursh) DC. (Rosales: Rosaceae), previously indicated as a host plant [34].

The few data available suggest that *G. ethela* flight is between the end of June and August [36,37]; this observation is supported by the emergence of adults in laboratory conditions (August and September). Carpentermoths usually are nocturnal, and they lay their eggs in plant crevices or under bark, where the newly hatched larva starts to mine the wood, completing the development in one to four years. However, larval biology is unknown for most of the *Givira* species [45]. Before pupation, the carpentermoth larva prepares a way out by chewing an exit hole up to the surface of the trunk. At adult emergence, the pupal exuvia remains on the gallery exit, protruding from the trunk. This behavior is present in *G. ethela* (this paper) as well as in other *Givira* species [39].

In this study, we also observed the association between *P. ficus* and *G. ethela* galleries. *Planococcus ficus* infestation was particularly prevalent in table grape trunks, especially at the Clovis site. Mealybugs are pests found in most of the grapevine production areas in the world and in high densities can reduce fruit quality and vine vigor, and their excreted honeydew can foul fresh marked grapes and promotes the development of molds, thus reducing fruit marketability [47,48]. Moreover, mealybug pest status is increased because most tested species are vectors of grape leafroll virus, which decreases vine vigor and crop size, as well as the quality of the produced wine [49,50].

Carpentermoth larvae are often involved in community ecology; for example, in forest ecosystems they have been reported to make shelters for other insects and promote access to plant sap that is involved in the attraction of other arthropods [39,51,52]. Here, we suggest a novel mutualistic association as *G. ethela* galleries may favor vineyard mealybugs by facilitating their access to the vine sap, protecting them from predators and parasitoids and offering shelter from environmental stresses and pesticide treatments; in turn, we note that the galleries were always free of mealybug honeydew that we assume the cossid feeds on. Similarly, *Crematogaster ashmeadi* Wheeler, 1932 (Hymenoptera: Formicidae) does not mine its own galleries but is often found inhabiting abandoned galleries of *Givira francesca* (Dyar, 1909) in North America and of other xylophagous insects [39]. In Japan, carpentermoth larvae are known to induce and maintain plant sap exudation from trunks through their wood boring activity, attracting other insects that feed on the plant sap [51,52].

Among practices applicable to develop an integrated pest management (IPM) approach is the proper identification of pest species, in this case *G. ethela*, and the development of monitoring tools to help determine pest control actions [19]. In other ecosystems, adult *Givira* species were successfully collected using blacklights, mercury vapor lights, and ultraviolet (UV) light traps, e.g., [40,53,54]. Traps baited with sex pheromones are another possible tool that could be developed, as mating disruption with sex pheromones has been used against other carpentermoth species [55,56,57]. Mass trapping has also been tested for the cossids *Zeuzera pyrina* (Linnaeus, 1761) and *C. cossus* [58,59,60]. Another case in the use of sex pheromones is for the cossid *Coryphodema tristis* (Drury, 1782) on *Eucalyptus nitens* (H. Deane and Maiden) Maiden in South Africa, where the large-scale mass trapping suppressed this pest [61]. Notably, the use of sticky UV-light traps in combination with sex pheromone traps successfully attracted *Z. pyrina* adults [58], where the light is attractive for both males and females. However, UV-light traps are a wide-spectrum attractants in respect to moths [62], and they may have considerable effects on non-target insects and even vertebrates, e.g., [63,64,65]. For these reasons, monitoring methods should include highly specific traps in order to minimize their impact on non-target species.

Finally, natural enemies could be another variable to take into account when considering carpentermoth control in vineyards [5]. In the present study, a *Digonogastra* species commonly emerged from *G. ethela* larvae; this group of braconids is known to parasitize lepidopteran larvae, e.g., [66,67,68] but further efforts are needed to understand *Digonogastra*’s biology and impact on *G. ethela* populations in vineyards.

## 5. Conclusions

This work reports *G. ethela* as a new wood boring pest of grapevines in California, and updates its distribution in the area. Pest identification comprising morphological and molecular features is a key tool for proper pest management. We report not only on this novel pest association but provide molecular coding data that may more rapidly provide identification of this pest should its geographic range expand. In a view of the IPM framework, tools against *G. ethela* should include the best applicable solutions, including all possible tasks such as the study of natural enemies or the application of pheromone traps for its control. To develop sustainable IPM programs, the geographic range and pest incidence must be better understood. For this to properly occur, methods to economically monitor *G. ethela* should be developed as well as a better understanding of its economic injury to the vine and relationship with *P. ficus*, which is perhaps the most important vineyard pest in California. The possibility to include more sustainable controls would include investigations of the pest’s sex pheromones for monitoring and control, as well as a better understanding of its natural enemies [19,20].

## Data Availability

Barcodes sequences are available in Supplementary Materials.

## Supplementary Materials

The following are available online at https://www.mdpi.com/2075-4450/12/3/239/s1, Table S1: Barcodes complete sequences of *Givira* species available in BOLD System [29] used in the maximum likelihood tree, Table S2: *Givira ethela* barcode details obtained from the analyzed samples.
