# Supplementary material for: Givira ethela (Neumoegen and Dyar, 1893) (Lepidoptera: Cossidae), A Previously Unidentified Pest on Vitis vinifera (L.)
Source: Insects. 2021 Mar 12;12(3):239. doi: 10.3390/insects12030239 (PMC7998413; doi:10.3390/insects12030239)
Supplement: Supplementary file 1 [file insects-12-00239-s001.pdf]

Table S1. Barcodes complete sequences of *Givira* species available in BOLD System [29] used in the Maximum likelihood tree.

Species	BOLD System number	County	State
Givira ethela (Neumoegen & Dyar, 1893)	BBLOC1217-11	Mono	California
	BBLOC1253-11	Mono	California
	BBLOC1283-11	Mono	California
Givira cornelia (Neumoegen & Dyar, 1893)	LTOL666-07	Mono	California
	CNCLA440-13	Grant	Washington
	CNCLA441-13	Grant	Washington
	CNCLA633-13	Walla Walla	Washington
	CNCLA634-13		British Columbia (Canada)
	CNCLA861-13	Baker	Oregon
Givira francesca (Dyar, 1909)	BBLOC023-11	Santa Rosa	Florida
	LOFLA048-06	Putnam	Florida
	LOFLA167-06	Putnam	Florida
	LOFLA254-06	Putnam	Florida
	LOFLA286-06	Putnam	Florida
	LOFLA300-06	Putnam	Florida
	LOFLA703-06	Putnam	Florida
	LOFLA705-06	Putnam	Florida
	LOFLA747-06	Putnam	Florida
	LOFLB105-06	Highlands	Florida
	LOFLB107-06	Highlands	Florida
	LOFLB148-06	Highlands	Florida
	LOFLB197-06	Highlands	Florida
	LOFLD050-07	Putnam	Florida
Givira anna (Dyar, 1898)	LOFLA206-06	Putnam	Florida
Givira lotta Barnes & McDunnough, 1910	CMAZA755-10	Cochise	Arizona
	CMAZA757-10	Cochise	Arizona
	IAWL998-09	Santa Cruz	Arizona
	LNAUS146-12	Cochise	Arizona
Givira minuta Barnes & McDunnough, 1910	LNAUS055-12	Cochise	Arizona
Givira arbeloides (Dyar, 1899)	BLPAA585-06		Guanacaste (Costa Rica)
	BLPDG488-09		Guanacaste (Costa Rica)
	BLPDH156-09		Guanacaste (Costa Rica)
	BLPEE1342-12		Guanacaste (Costa Rica)
	BLPEE428-12		Guanacaste (Costa Rica)
	BLPEE547-12		Guanacaste (Costa Rica)
	MHMYL2585-11		(Costa Rica)
	MHMYL2586-11		(Costa Rica)
	MHMYL4342-16		Guanacaste (Costa Rica)
	MHMYL4343-16		Guanacaste (Costa Rica)
	MHMYL4344-16		Guanacaste

			(Costa Rica)
	MHMYL4345-16		Guanacaste (Costa Rica)
	MHMYL4346-16		Guanacaste (Costa Rica)
	MHMYL4347-16		Guanacaste (Costa Rica)
	MHMYL4348-16		Guanacaste (Costa Rica)
	MHMYL4349-16		Guanacaste (Costa Rica)
	MHMYL4432-16		Guanacaste (Costa Rica)
	MHMYL4547-16		Guanacaste (Costa Rica)
	MHMYL4691-16		Guanacaste (Costa Rica)
	MHMYL4692-16		Guanacaste (Costa Rica)
	MHMYL4869-16		(Costa Rica)
	MHMYL4870-16		Guanacaste (Costa Rica)
	MHMYL4872-16		Guanacaste (Costa Rica)
	MHMYL4873-16		Guanacaste (Costa Rica)
	MHMYN6927-14		Guanacaste (Costa Rica)
Givira theodori (Dyar, 1893)	LNAUS047-12	Brewster	Texas
	TXLEP1035-20	Edwards	Texas
	TXLEP205-15	Edwards	Texas
Hypopta palmata Barnes & McDunnough, 1910	LNAUS078-12	Pima	Arizona
	LNAUS079-12	Pima	Arizona
	LNAUS080-12	Pima	Arizona
Cossus cossus (Linnaeus, 1758)	FBLMV612-09	Oberpfalz	Bavaria (Germany)
	FBLMX240-11	Oberpfalz	Bavaria (Germany)

Table S2. *Givira ethela* barcode details obtained from the analyzed samples.

Specimen	Collection data	Barcode
Givira sp. 1 (Adult)	Clovis, Fresno County, CA; C. K.M. Daane; 3.IV.2019	TAAGTCTTCTAATTCGAGCCGAATTAGGTAATCCAG-<br>GATCCTTAATTGGAGAC-<br>GATCAAATCTATAATACTATTGTTACAGCCCATGCTTTTATT<br>ATAATCTTTTTCATAGTTATACCAATTATAATTGGAGGAT-<br>TTGGAAATTGACTT-<br>GTGCCCCTAATATTAGGGGCACCAGACATAGCATTCCCAC<br>GAATAAATAACATAAGATTTT-<br>GACTTCTTCCCCCCTCCCTACTCCTTTTAATTTCAAGAA-<br>GAATCGTAGAAAACGGAGCAGGTACAGGATGAACAGTTT<br>ATCCCCCCCTTTCCTCTAATATTGCCCATAGAG-<br>GAACATCCGTAGACTTAG-<br>CAATTTTTTCCCTCCACTTAGCTGGAATTTCATCTATTCTAG<br>GAGCTGTAAATTTTATTAC-<br>CACAATTATTAACATAAAACCCCATAAAA-<br>TATCCTTCGATCAAATACCACTATTTGTTTGAGCAGTAGGA<br>ATTACAGCCTTATTATTACTCCTCTCTCTACCAGTATTAG-<br>CAGGAGCCATCAC-<br>CATACTTCTAACTGATCGAAATATTAATACATCATTTTTCG<br>ACCCCGCTGGTGGGGGAGACCCTATCCTATACCAACAC-<br>TTATTTTGATTTTTTGGTC
Givira sp. 2 (Adult)	Clovis, Fresno County, CA; C. D. Saccini; 12.XI.2018. p. m. 1733 TG	TAAGTCTTCTAATTCGAGCCGAATTAGGTAATCCAG-<br>GATCCTTAATTGGGGAC-<br>GATCAAATCTATAATACTATTGTTACAGCCCATGCTTTTATT<br>ATAATCTTTTTCATAGTTATACCAATTATAATTGGAGGAT-<br>TTGGAAATTGACTT-<br>GTGCCCCTAATATTAGGGGCACCAGACATAGCATTCCCAC<br>GAATAAATAACATAAGATTTT-<br>GACTTCTTCCCCCCTCCCTACTCCTTTTAATTTCAAGAA-<br>TAATCGTAGAAAACGGAGCAGGTACAGGATGAACAGTTTA<br>TCCCCCCCTTTCCTCTAATATTGCCCATAGAGGAACATCCG-<br>TAGACTTAG-<br>CAATTTTTTCCCTCCACTTAGCTGGAATTTCATCTATTCTAG<br>GAGCTGTAAATTTTATTAC-<br>CACAATTATTAACATAAAACCCCATAAAA-<br>TATCCTTCGATCAAATACCACTATTTGTTTGAGCAGTAGGA<br>ATTACAGCCTTATTATTACTCCTCTCTCTACCAGTATTAG-<br>CAGGAGCCATCAC-<br>CATACTTCTAACTGATCGAAATATTAATACATCATTTTTCG<br>ACCCCGCTGGTGGGGGAGACCCTATCCTATACCAACAC-<br>TTATTTTGATTTTTTGGTC
Givira sp. 3 (Adult)	Clovis, Fresno County, CA; C. D. Saccini; 12.XI.2018. p. m. 1736 TG	TAAGTCTTCTAATTCGAGCCGAATTAGGTAATCCAG-<br>GATCCTTAATTGGAGAC-<br>GATCAAATCTATAATACTATTGTTACAGCCCATGCTTTTATT<br>ATAATCTTTTTCATAGTTATACCAATTATAATTGGAGGAT-<br>TTGGAAATTGACTT-<br>GTGCCCCTAATATTAGGGGCACCAGACATAGCATTCCCAC<br>GAATAAATAACATAAGATTTT-<br>GACTTCTTCCCCCCTCCCTACTCCTTTTAATTTCAAGAA-<br>GAATCGTAGAAAACGGAGCAGGTACAGGATGAACAGTTT<br>ATCCCCCCCTTTCCTCTAATATTGCCCATAGAG-<br>GAACATCCGTAGACTTAG-<br>CAATTTTTTCCCTCCACTTAGCTGGAATTTCATCTATTCTAG<br>GAGCTGTAAATTTTATTAC-<br>CACAATTATTAACATAAAACCCCATAAAA-<br>TATCCTTCGATCAAATACCACTATTTGTTTGAGCAGTAGGA<br>ATTACAGCCTTATTATTACTCCTCTCTCTACCAGTATTAG-<br>CAGGAGCCATCAC-<br>CATACTTCTAACTGATCGAAATATTAATACATCATTTTTCG<br>ACCCCGCTGGTGGGGGAGACCCTATCCTATACCAACAC-<br>TTATTTTGATTTTTTGGTC
Givira sp. 4 (Larva)	Clovis, Fresno County, CA; C. D. Saccini; 12.XI.2018	TAAGTCTTCTAATTCGAGCCGAATTAGGTAATCCAG-<br>GATCCTTAATTGGGGAC-<br>GATCAAATCTATAATACTATTGTTACAGCCCATGCTTTTATT

	ATAATCTTTTTCATAGTTATACCAATTATAATTGGAGGAT-<br>TTGGAAATTGACTT-<br>GTGCCCCTAATATTAGGGGCACCAGACATAGCATTCCCAC<br>GAATAAATAACATAAGATTTT-<br>GACTTCTTCCCCCCTCCCTACTCCTTTTAATTTCAAGAA-<br>GAATCGTAGAAAACGGAGCAGGTACAGGATGAACAGTTT<br>ATCCCCCCCTTTCCTCTAATATTGCCCATAGAG-<br>GAACATCCGTAGACTTAG-<br>CAATTTTTTCCCTCCACTTAGCTGGAATTTCATCTATTCTAG<br>GAGCTGTAAATTTTATTAC-<br>CACAATTATTAACATAAAACCCCATAAAA-<br>TATCCTTCGATCAAATACCACTATTTGTTTGAGCAGTAGGA<br>ATTACAGCCTTATTATTACTCCTCTCTCTACCAGTATTAG-<br>CAGGAGCCATCAC-<br>CATACTTCTAACTGATCGAAATATTAATACATCATTTTTCG<br>ACCCCGCTGGTGGGGGAGACCCTATCCTATACCAACAC-<br>TTATTTTGATTTTTTGGTC
